# Improving the Mechanical Properties of Cu-15Ni-8Sn Alloys by Addition of Titanium

**DOI:** 10.3390/ma10091038

**Published:** 2017-09-06

**Authors:** Chao Zhao, Weiwen Zhang, Zhi Wang, Daoxi Li, Zongqiang Luo, Chao Yang, Datong Zhang

**Affiliations:** 1 Guangdong Key Laboratory for Processing and Forming of Advanced Metallic Materials, South China University of Technology, Guangzhou 510640, China; meczhao@mail.scut.edu.cn (C.Z.); wangzhi@scut.edu.cn (Z.W.); 201610100610@mail.scut.edu.cn (D.L.); mezqluo@scut.edu.cn (Z.L.); cyang@scut.edu.cn (C.Y.); dtzhang@scut.edu.cn (D.Z.); 2 School of Mechanical and Automotive Engineering, South China University of Technology, Guangzhou 510640, China

**Keywords:** Cu-15Ni-8Sn alloy, titanium, mechanical properties, grain refinement, discontinuous precipitation

## Abstract

The effect of Ti addition on the microstructure and mechanical properties of Cu-15Ni-8Sn alloys was investigated. Optical microscopy (OM), scanning electronic microscopy (SEM), and transmission electron microscopy (TEM) were used to determine grain size and distribution of the second phases in the alloys. The results indicate that the tensile properties of Cu-15Ni-8Sn alloys are improved significantly with Ti addition. Tensile elongation increased from 2.7% for the alloy without Ti to 17.9% for the alloy with 0.3% Ti, while tensile strength was maintained and even increased from 935 MPa to 1024 MPa. The improvement of the mechanical properties of Cu-15Ni-8Sn alloys by the addition of Ti is attributed to the grain refinement and suppression of discontinuous precipitation during heat treatment.

## 1. Introduction

Cu-Ni-Sn alloys are considered as an attractive substitute for Cu-Be alloys, and have been widely applied to the manufacture of switches, connectors, and spring components in the electronic industries [1,2,3]. Among Cu-Ni-Sn systems, Cu-15Ni-8Sn (known as C72900) has the highest strength. Due to its excellent strength and high wear resistance, Cu-15Ni-8Sn alloy is also an ideal candidate for high-performance bearings and highly wear-resistant components for aerospace and mechanical systems [4,5].

It has been found that Cu-15Ni-8Sn alloy can reach a tensile strength in excess of 1000 MPa by suitable thermomechanical treatment [6]. However, such thermomechanical treatment generally results in disappointing low ductility [7,8,9]. Cribb reported that the tensile strength of the Cu-15Ni-8Sn alloy reached 1140 MPa, but the elongation was only 3% [10]. Discontinuous precipitation has been considered to be one of the main reasons for the loss of ductility, which appeared at grain boundaries in the aging process [11,12]. Many works have attempted to suppress discontinuous precipitation by optimizing thermomechanical treatment. The initial work on thermomechanical processing of Cu-Ni-Sn alloy was done by J.T Plewes [3], showing that ductility response is a consequence of competitive balance between spinodal and discontinuous transformation. Lefevre [13,14] pointed out that spinodal hardening in Cu-15Ni-8Sn alloy was limited by the discontinuous precipitation. Despite a drop of the yield strength, the ductility still remained low in the overaging process. 

On the other hand, it has been confirmed that the addition of trace elements can effectively suppress the discontinuous precipitation reaction. Insoluble fine particles were observed in the matrix and the grain boundaries of the Cu-10Ni-8Sn alloys with different addition of Si, Al, or Cr. These particles occupied the nucleation sites to suppress the nucleation of the discontinuous precipitation and retarded the migration of the advancing boundaries of the cell and matrix by Zener′s pining effect to inhibit the growth of the cell and refine the grains; meanwhile, the additions barely diminished the maximum hardness of the alloys [15,16,17]. In the Cu-15Ni-8Sn alloy with the addition of silicon, the nucleation and growth of discontinuous precipitation are suppressed, caused by the formation of Ni_2_Si and Ni_3_Si particles [18]. The addition of Nb, Ta, V, or Fe also remarkably increased ductility of the Cu-15Ni-8Sn alloy with suitable cold work and aging treatment [8,9].

It has been found that the addition of Ti also has the effect of suppressing discontinuous precipitation and grain refinement in the Cu-10Ni-8Sn alloy, owing to the formation of insoluble particles [17]. As discussed above, Cu-15Ni-8Sn alloys with high strength have an important technical application; however, their low ductility limits their wider application. It is expected that the mechanical properties of the Cu-15Ni-8Sn alloy could be improved by the suppression of discontinuous precipitation and grain refinement by the addition of Ti. Therefore, in the present work, the effect of Ti addition on the high strength Cu-15Ni-8Sn alloy was studied; the ductility of the alloys in the present work was effectively improved, maintaining an ultra-high level of strength.

## 2. Experimental Procedure

### 2.1. Materials and Processing

Cu-15Ni-8Sn alloy ingots with different Ti contents were prepared by an intermediate frequency induction furnace. The chemical compositions of these alloys are listed in Table 1. The cast ingots were subsequently homogenization treated at 840 °C for 8 h. The hot extrusion process was utilized to fabricate a rod with diameter of 12 mm at an extrusion ratio of 17:1. After solution treatment at 820 °C for 1 h, the extruded rods were quenched into water and then aged isothermally at 400 °C for 4 h.

### 2.2. Microstructure Evaluation

**Figure 1:**
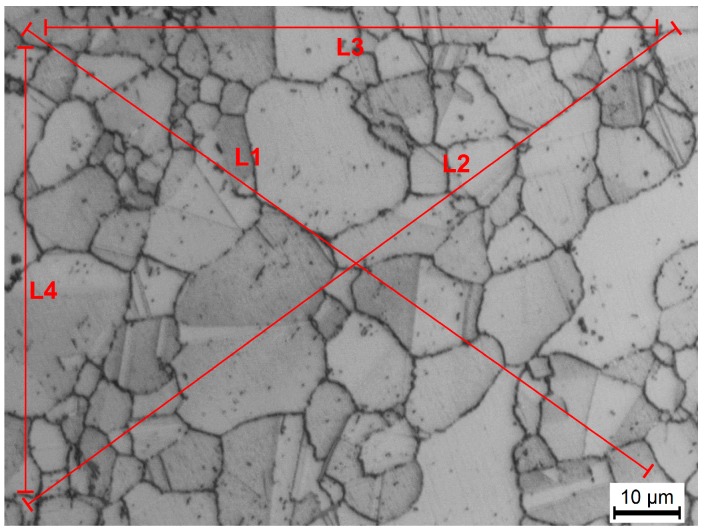
The representative optical microscopy (OM) image of grain size determination by the linear intercept method.

The microstructure of each specimen was characterized by optical microscopy (OM, Leika, Microsystems GmbH, Wetzlar, Germany), scanning electronic microscopy (SEM, FEI, Hillsboro, OR, USA), and transmission electron microscopy (TEM, FEI, Hillsboro, OR, USA). Grain structure and fracture surfaces were examined with a LEICA/DMI 5000M optical microscope (Leika, Microsystems GmbH, Wetzlar, Germany) and a FEI NONA430 scanning electron microscope. The mean grain size was obtained from 20 random areas of each specimen by the linear intercept method based on the observation of OM. The representative OM image of determination of grain size by the linear intercept method is shown in Figure 1. The specimens for microstructure observation were prepared by polishing and then etching in a solution of 5 g FeCl_3_ + 10 mL HCl + 100 mL H_2_O. Transmission electron microscope observation was performed on a FEI TECNAI G2 S-TWIN F20 (FEI, Hillsboro, OR, USA). TEM samples were prepared by twin-jet electro-polishing method in 95% alcohol and 5% perchloric acid at −25 °C.

### 2.3. Resistivity Measurement

Distribution of atoms was estimated by monitoring the absolute resistance change in different treatment conditions. The electrical resistivity was measured at room temperature by a PPMS-9 resistivity analyzer (Qyantum Design Inc., Design Inc., San Diego, CA, USA). The average resistivity was calculated from at least five independent values for each specimen.

### 2.4. Mechanical Properties Testing

The aged rods were cut into cylindrical tensile samples with a gauge section of 5 mm in diameter and 25 mm in length according to Chinese GB/T 228-2002. Tensile tests were performed on a SANS CMT5105 material test machine (SANS, Shenzhen, Guangdong, China). Three replicates were used to establish the mechanical property data.

## 3. Results and Discussion

### 3.1. Microstructures

**Figure 2:**
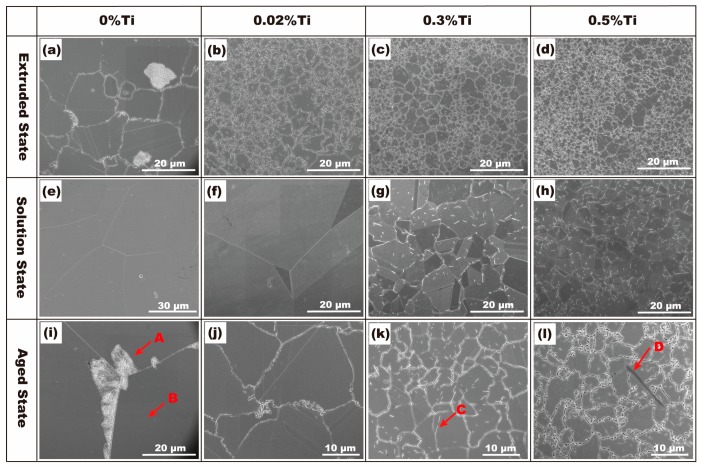
SEM micrographs of alloys with different contents of Ti in different conditions: (**a**–**d**) Hot-extruded state; (**e**–**h**) Solid solution treatment at 820 °C for 1 h; (**i**–**l**) Aging treatment at 400 °C for 4 h.

Figure 2 indicates the microstructures of the alloys with different Ti contents in different conditions: hot-extrusion, solid solution treatment at 820 °C for 1 h, and aged at 400 °C for 4 h. For the aged alloy without the addition of Ti, coarse grains and discontinuous precipitation in the grain boundaries (arrow A in Figure 2i) were clearly observed. For the aged alloys with the addition of 0.02% Ti, discontinuous precipitation was suppressed (Figure 2j) and no Ti-rich precipitate was observed in solutionized alloy (Figure 2f). When the Ti content was equal to or more than 0.3%, needle-like precipitates were observed (arrow C in Figure 2k) and the discontinuous precipitation colony was not found in the aged alloys. It was found that the needle-like precipitates were formed in the solidification process. These precipitates could be retained in specimens after solution treatment at 820 °C for 2 h (Figure 2g,h). With the addition of 0.5% Ti to the alloy, the amount of the precipitates increased significantly, and a small quantity of precipitates showed coarsening after aging treatment (arrow D in Figure 2l).

**Figure 3:**
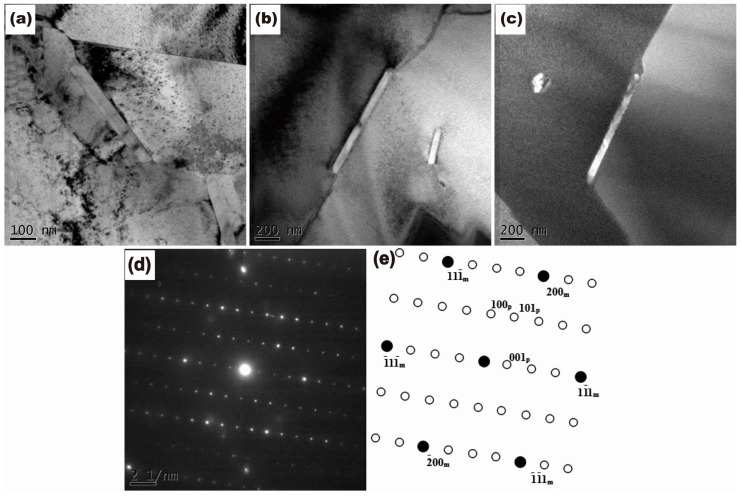
TEM images of Ni_3_Ti precipitates distributed in the grain boundary in 0.5% Ti alloys: (**a**) hot-extruded alloy; (**b**) solid solution alloy; (**c**) aged alloy; (**d**) SADP of the Ni_3_Ti phase in 0.5% Ti aged alloy; (**e**) Schematic of the SADP in (d).

Energy-dispersive X-ray (EDX) results of the chemical composition of second phases in the alloys are listed in Table 2. The EDX results show that the needle-like precipitates are Ni_3_Ti phase (arrow C in Figure 2k and arrow D in Figure 2l), where the ratio of Ni and Ti is approximately 3:1, which is further confirmed by the SADP. Figure 3 shows the results of TEM observation of the alloy with the addition of 0.5% Ti. Based on the EDX data, analysis of SADP reveals that the Ti-rich precipitates were determined to be Ni_3_Ti phase.

**Figure 4:**
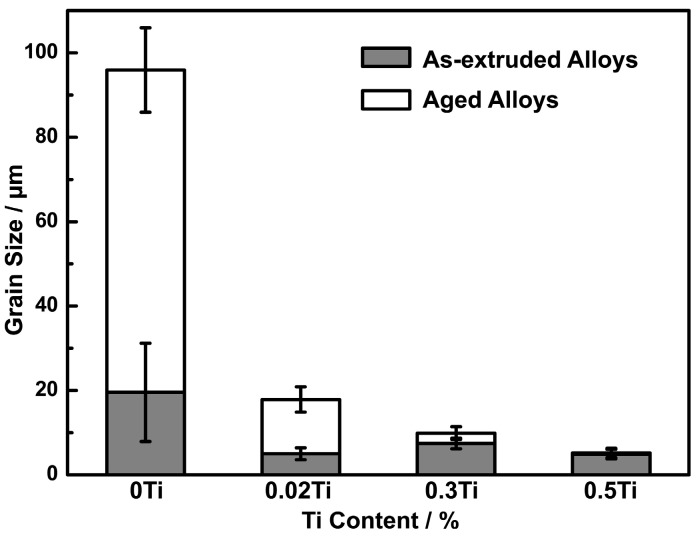
Grain size of the as-extruded and aged alloys.

Figure 4 shows the change of grain size of the alloys in different treatment states. It can be seen that the grain size of the aged alloys decreased with increasing Ti content, from 95.9 µm to 5.2 µm. The grain refinement effect induced by Ti addition is attributed to two factors. Firstly, the addition of Ti stimulates recrystallization nucleation in hot-extrusion process. In the process of hot-extrusion, Ni_3_Ti precipitates enhance the dislocation density, which promotes recrystallization nucleation [19,20] (Figure 3a). Secondly, the growth of grains during heat treatment is inhibited by Ti addition. For the alloys with addition of 0.3% Ti and 0.5% Ti, Ni_3_Ti precipitates distributed in grain boundaries can retard the grain boundary migration in the process of solid solution treatment (Figure 3b), which decreases the grain growth rate remarkably.

**Figure 5:**
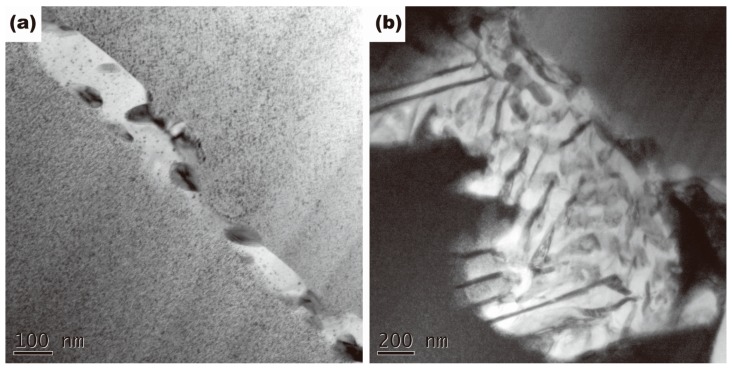
TEM images of the morphology of discontinuous precipitation in 0.02% Ti alloy aged at 400 °C for 4 h: (**a**) Initiation of discontinuous precipitation in grain boundaries; (**b**) Typical colony of discontinuous precipitation.

**Figure 6:**
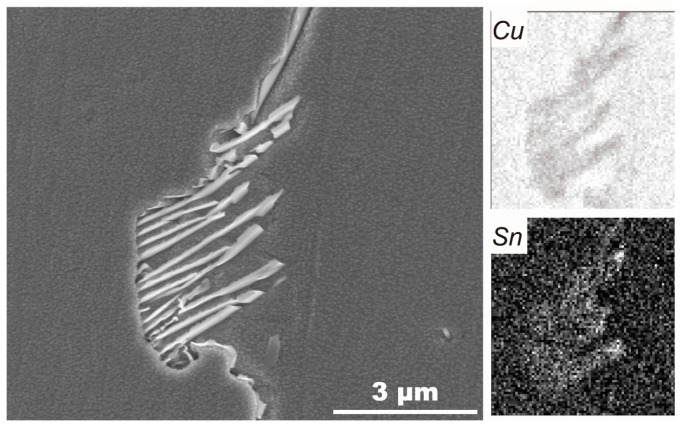
Energy-dispersive X-ray (EDX) map of Cu and Sn taken from the second phase in 0.02% Ti alloy aged at 400 °C for 4 h.

The discontinuous precipitations are clearly observed at grain boundaries in the alloys without Ti and with 0.02% Ti (Figure 2i and Figure 5), which are constituted by γ lamellae (DO_3_ (Cu_x_,Ni_1-x_)_3_Sn) and depleted α lamellae [21]. The EDX map of Cu and Sn in the colony of discontinuous precipitation in 0.02% Ti alloy is presented in Figure 6. Figure 5a depicts the initiation process of discontinuous precipitation at the grain boundary. The typical lamellar structure of discontinuous precipitation is shown in Figure 5b.

The initiation process of discontinuous precipitation of the Cu-15Ni-8Sn alloys can be illustrated by the pucker mechanism [22]. In the initiation process of discontinuous precipitation, it is necessary that some positions of the grain boundary are deformed into shapes with special small angle to form the nucleation position of discontinuous precipitation (Figure 5a). In the previous work [15,16,17], it was reported that insoluble particles occupy the sites of discontinuous precipitation, which is contributed to suppression of the formation of discontinuous precipitation. As Figure 3c shows, no facet was found at the grain boundary near Ni_3_Ti phase in the alloys with addition of 0.3% Ti and 0.5% Ti. Therefore, it is demonstrated that the effect of Ni_3_Ti phase on grain boundary is not only to hinder the grain boundary migration, but also to suppress the puckering of grain boundary. The number of nucleation sites of discontinuous precipitation obtained by puckering of grain boundary was reduced. As a result, the addition of Ti had an effect on the suppression of the initiation process of discontinuous precipitation. 

The effect of Ti addition on the growth of discontinuous precipitation can be discussed by the following formula of the growth rate of discontinuous precipitation [23].

(1) G=M×P

where G corresponds to the growth rate of the cell, M is the mobility of frontier interface of the cell, P is the driving force of the cell growth. In case of the alloys with Ti addition, Ni_3_Ti phase in the grain boundary produces a back-driving force ($P_{P}$) and decreases M due to the suppression of migration of the frontier interface. Consequently, the nucleation and growth of discontinuous precipitation are both suppressed by the addition of Ti. In recent years, it has become widely accepted that the grain boundary “complexion” transitions could also lead to discontinuous changes in mobility and diffusivity of grain boundary [24,25]. It is reasonable to speculate that the addition of Ti elements changes grain boundary complexion, and thus intergranular discontinuous precipitation might be suppressed [26,27].

In order to clarify the solid solubility of Ti in the Cu-15Ni-8Sn alloy and the effect of Ti addition on γ phase, the distributions of Ni, Sn, and Ti atoms were determined. Table 3 lists the values of electrical conductivity used to calculate the distributions of those solute atoms in the base and 0.3% Ti alloys. E0 and E1 are the relative electrical conductivity of the alloys after and before aging at 400 °C for 4 h, respectively. E2 is the relative electrical conductivity of the alloys after solution treatment at 960 °C for 3 h. On the basis of the microstructure observations, γ phase was dissolved in matrix but Ni_3_Ti phase still existed in 0.3% Ti alloy after solution treatment at 820 °C for 1 h (Figure 2g). After solution treatment at 960 °C for 3 h, Ni_3_Ti phase was entirely dissolved in 0.3% Ti alloy. The change in the electrical resistivity Δρ of the alloys on different treatment state can be described as [28]:

(2) $$\Delta \rho =\Delta \rho_{G.B.}+\Delta \rho_{DIS}+\Delta \rho_{SOL}$$

$\Delta \rho_{G.B.}$, $\Delta \rho_{DIS}$, and $\Delta \rho_{SOL}$ are the resistivity changes contributed by the changes in grain boundaries, dislocation density, and solute atom concentration in the alloys, respectively. For Cu-15Ni-8Sn alloy with addition of 0.3% Ti, the effect of the changes in grain boundary density is considered to be negligibly small, owing to little change in the grain size of the 0.3% Ti alloy. According to a previous study [29], $\Delta \rho_{DIS}/\Delta N$ of copper is about 2 × 10^−16^ nΩm^3^, which is a significantly smaller contribution to resistivity than the resistivity contribution of solute atoms of Ni, Sn, and Ti. Thus, the changes in resistivity primarily contributed to the changes in solute atom concentration. The values of relative electrical conductivity are calculated from the formula:

(3) $$E=\rho_{0}\times 100/\rho$$

Here, $\rho_{0}=17.24\;n\Omega m$. In case of 0.3% Ti alloy, the difference between E2 and E1 was mainly caused by precipitation of Ni_3_Ti phases, and precipitation of γ phases led to the difference between E1 and E0. The data used to calculate the atomic distributions are reported in Komatsu’s [30] study: $\Delta \rho_{Ni}=12.2\;n\Omega m/\mathrm{at}\%$, $\Delta \rho_{Sn}=28.8\;n\Omega m/\mathrm{at}\%$, $\Delta \rho_{Ti}=113.3\;n\Omega m/\mathrm{at}\%$.

**Figure 7:**
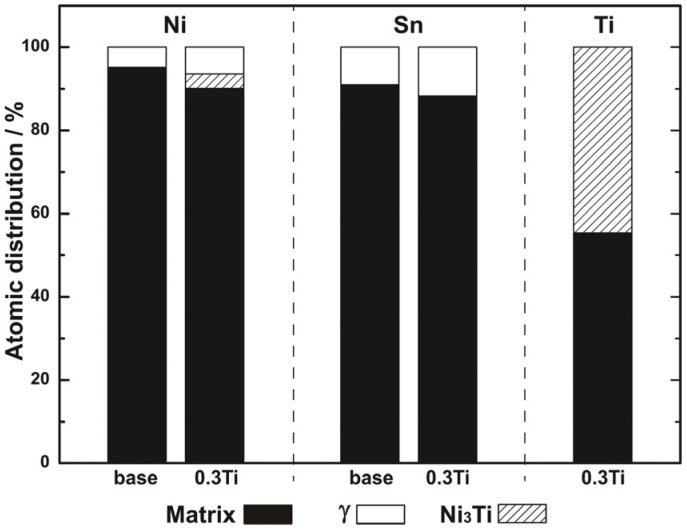
The distribution of Ni, Sn, and Ti atoms among the matrix, γ phases, and Ni_3_Ti precipitates in base and 0.3% Ti alloys aged at 400 °C for 4 h.

Figure 7 indicates the distributions of Ni, Sn, and Ti atoms in matrix, γ phase, and Ni_3_Ti precipitation. In case of 0.3% Ti alloy, approximately 0.177% Ti was dissolved in the α (Cu) matrix, and the remaining Ti formed the Ni_3_Ti phase. The solution of Ti in the matrix not only played a role in solid-solution strengthening, but also decreased solid solubility of Tin in the matrix, resulting in promotion of the precipitation of γ phase. The calculation result corresponds well with the result that no Ni_3_Ti precipitates are observed by TEM observation of the aged 0.02% Ti alloy.

### 3.2. Mechanical Properties

**Figure 8:**
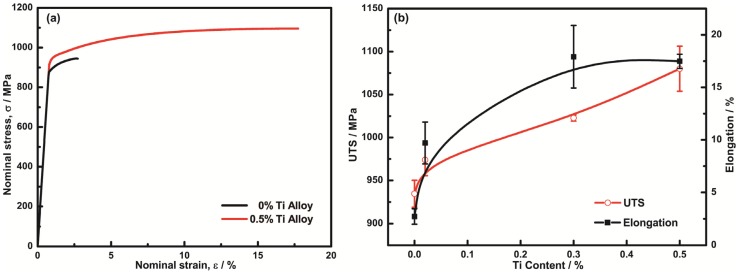
Mechanical properties of alloys with different contents of Ti: (**a**) Nominal stress–strain curves of 0% Ti and 0.5% Ti alloys; (**b**) Ultimate tensile strength (UTS) and tensile elongation of four alloys after aging at 400 °C for 4 h.

Figure 8a shows the nominal stress–strain curves of 0% Ti and 0.5% Ti alloys. It is demonstrated that the addition of Ti had the effect of improving the plasticity and strength of the alloys. Figure 8b depicts the mechanical properties of the aged alloys with different contents of Ti, indicating that both tensile strength and elongation were improved by the addition of Ti. When Ti content increased from 0 to 0.3%, the alloys exhibited significantly increased tensile elongation from 2.7 to 17.9%; meanwhile, the tensile strength increased from 935 MPa to 1024 MPa. Furthermore, the tensile strength was increased to 1080 MPa for the alloy with 0.5 Ti addition while the tensile elongation was slightly decreased to 17.5%. 

The increased strength is largely attributed to grain boundary strengthening and solid-solution strengthening. As shown above, the grain size of the alloys was significantly reduced by the addition of Ti—from 95.9 µm for the alloy without Ti to 5.2 µm for the alloy with 0.5% Ti. Therefore, the grain boundary strengthening may play an important role on strengthening according to the Hall–Petch relationship [31]. The solid-solution strengthening also helped to increase the strength. It is shown that with 0.3% Ti addition, 0.177% Ti was dissolved in the matrix.

**Figure 9:**
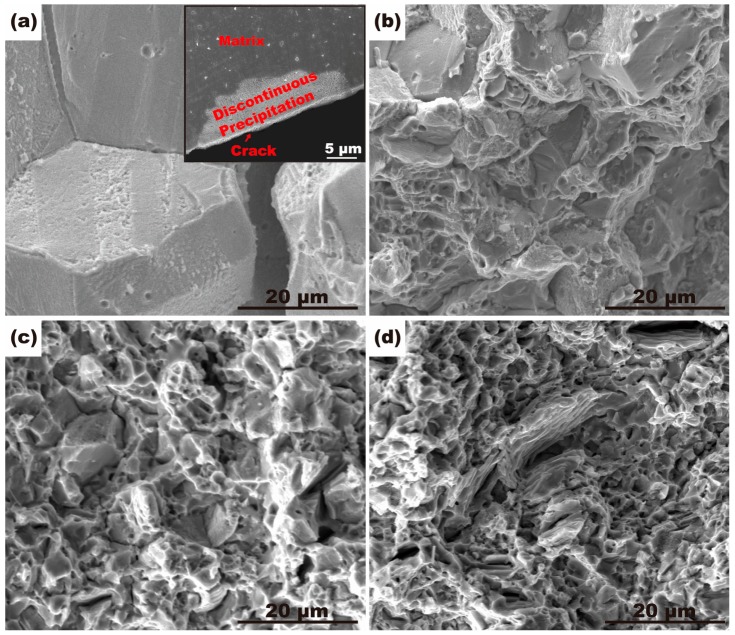
SEM morphologies of the fracture surfaces of the alloys with different contents of Ti: (**a**) 0% Ti, the insert image shows the tensile fracture surface after polishing; (**b**) 0.02% Ti; (**c**) 0.3% Ti; (**d**) 0.5% Ti.

The improved ductility of the alloys with the addition of Ti mainly contributed to the grain refinement and the suppression of discontinuous precipitation. As shown in Figure 9a, the morphology of discontinuous precipitation at the grain boundaries would be highly conducive to become the initiation site of intergranular cracks which reduce the stability of the grain boundary [22]. Therefore, the suppression effect of the addition of Ti on the discontinuous precipitation at the grain boundaries plays a significant role in the improvement of the ductility of the alloys.

Figure 9 shows the fracture surface morphology of the alloys aged at 400 °C for 4 h. In the case of the alloy without Ti addition (Figure 9a), intergranular fracture dominated. The coarse grains and flat grain boundaries make the cracks easy to propagate along grain boundaries. The insert in Figure 9a shows the longitudinal section of the tensile-fractured aged alloy without Ti, indicating that the colony of discontinuous precipitation was observed at the edge of the crack. Figure 9b–d depict that typical quasi-cleavage fracture with dimples and tear ridges appeared in the alloys with the addition of Ti. With increase of Ti addition, the amount of dimples increased and cleavage surface decreased. However, since the coarse Ni_3_Ti precipitates are pulled out, the hollows appear in the 0.5% Ti alloy. The decrease of elongation of the 0.5% Ti alloy is considered to be contributed by these hollows.

**Figure 10:**
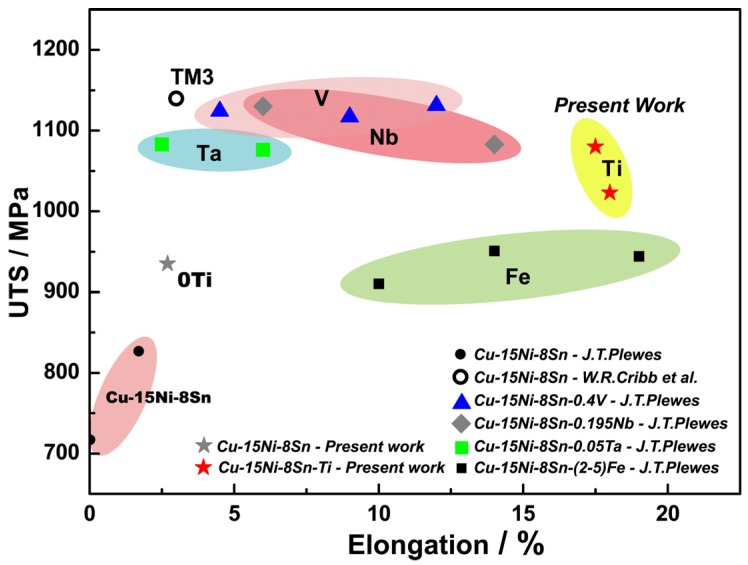
Comparison of mechanical properties of Cu-15Ni-8Sn alloys with the addition of Ti and other elements.

The tensile strength and elongation values of Cu-15Ni-8Sn alloys with Ti addition and other designed Cu-15Ni-8Sn alloys are summarized in Figure 10 [8,9,10]. Adding the appropriate amount of trace elements is an effective way to improve mechanical properties of the Cu-15Ni-8Sn alloys. Compared with additions of Nb, V, Fe, or Ta, the ductility of the alloy in the present work was improved effectively while maintaining an ultra-high level of strength by the addition of titanium.

## 4. Conclusions

In the present work, the effects of Ti addition on the microstructure and mechanical properties of Cu-15Ni-8Sn alloys were investigated. The following conclusions can be drawn:

(1) Cu-15Ni-8Sn alloys with the addition of Ti possess an excellent combination of strength and ductility. Compared to the alloy without Ti, the tensile strength and the elongation of the alloy with 0.3% Ti increased from 935 MPa to 1024 MPa and from 2.7% to 17.9%, respectively.
(2) The improvement of mechanical properties by the addition of Ti contributed to grain refinement and the suppression of discontinuous precipitation. By the addition of Ti, Ni_3_Ti phase was formed in the solidification process, which was undissolved in the following solution treatment. Ni_3_Ti phase had a pining effect on grain boundary migration in the process of solution treatment, and thus resulted in grain refinement. Ti addition also had a suppressive effect on discontinuous precipitation, owing to a reduction of nucleation sites of discontinuous precipitation.

## Figures and Tables

**Table 1:** Chemical composition of the Cu-15Ni-8Sn alloys with different Ti contents.

Alloy Designation	wt./%			
	Ni	Sn	Ti	Cu
Cu-15Ni-8Sn	15.03	8.12	-	balance
Cu-15Ni-8Sn-0.02Ti	15.03	8.14	0.02	balance
Cu-15Ni-8Sn-0.3Ti	15.23	8.36	0.32	balance
Cu-15Ni-8Sn-0.5Ti	14.96	8.02	0.53	balance

**Table 2:** EDX analysis results for the position indicated by the arrow A, B, C, and D in Figure 2.

	at./%			
	Cu	Ni	Sn	Ti
A	47.81	40.29	11.90	-
B	80.35	15.56	4.09	-
C	40.23	45.95	4.02	9.80
D	7.75	68.40	2.98	20.87

**Table 3:** Values of the electrical conductivity of the base and 0.3% Ti alloys.

Alloy	Electrical Conductivity (pct IACS ^1^)		
	E0	E1	E2
Base	41.20	27.29	-
0.3Ti	49.72	27.19	18.57

^1^ Relative Conductivity according to International Annealed Copper Standard.
